# Supplementary material for: High-resolution CTCF footprinting reveals impact of chromatin state on cohesin extrusion
Source: Nat Commun. 2025 May 15;16:4506. doi: 10.1038/s41467-025-57775-w (PMC12081859; doi:10.1038/s41467-025-57775-w)
Supplement: Supplementary file 1 — Supplementary Information [file 41467_2025_57775_MOESM1_ESM.pdf]

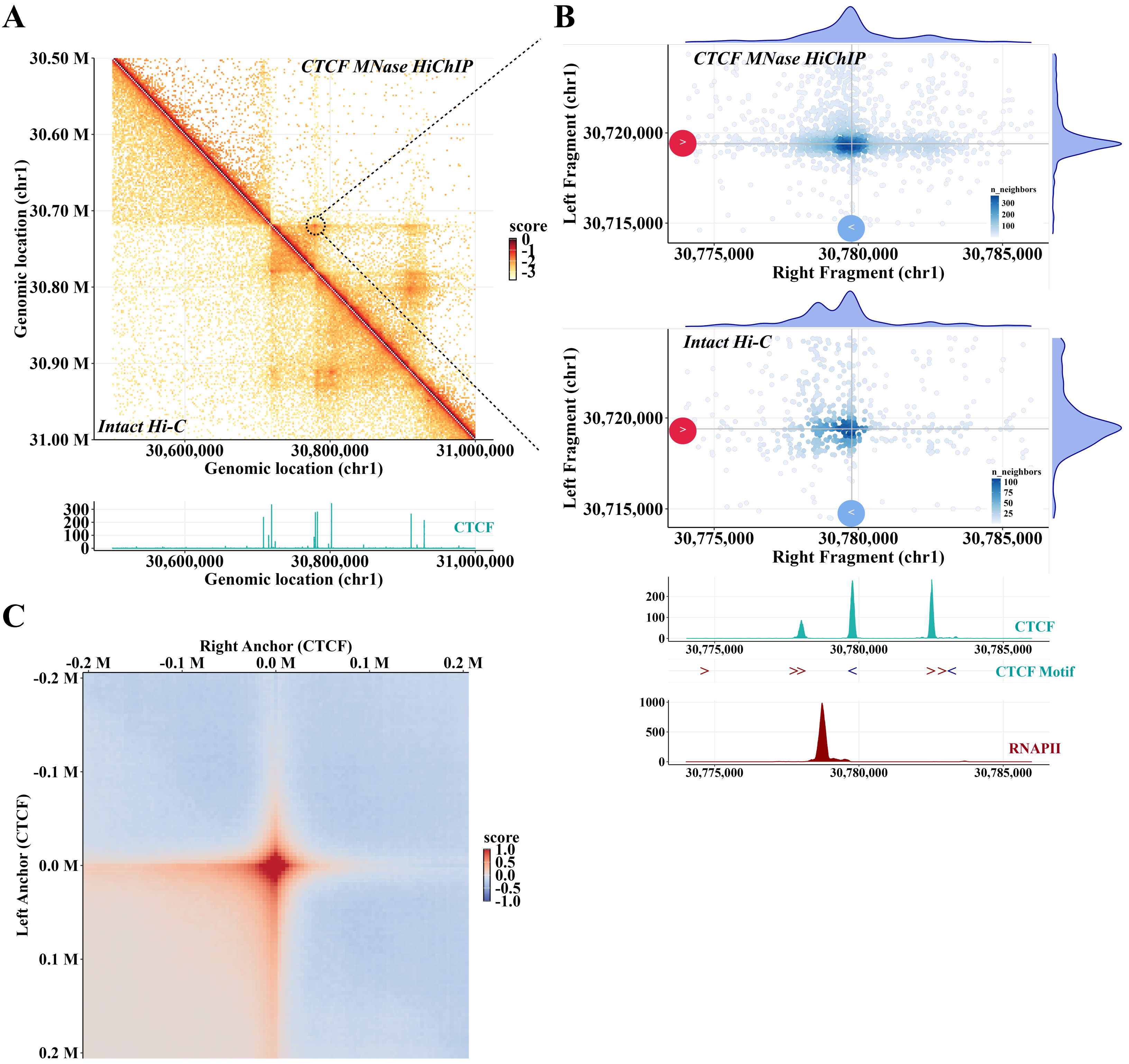

Supplementary Fig. 1. Comparison of K562 CTCF MNase HiChIP to K562 Intact Hi-C. A K562 CTCF MNase HiChIP (above diagonal) and K562 Intact Hi-C (GSE237898, below diagonal) balanced contact maps (log10 scale, 2kb resolution) for a region on chromosome one with CTCF ChIP-Seq annotation below (ENCFF168IFW). **B** Marginal densities and scatterplots colored by number of neighbors of left fragments vs right fragments (5' end of the read) for the loop circled in panel **(A)** between two convergent CTCF motifs: chr1:30,719,390-30,719,408 (+), chr1: 30,779,763-30,779,781 (-). The red (>) and blue (<) symbols respectively denote CTCF (+) and CTCF (-) motifs. CTCF ChIP-Seq (ENCFF168IFW), CTCF motifs, and RNAPII ChIP-Seq (ENCFF914WIS) annotations for the right anchor are shown below the scatterplots. **C** Metaplot of Intact Hi-C contacts aggregated over chromosome one CTCF HiChIP loops (n = 3645 loops, resolution = 4kb, linear scale, detrended contacts. Red represents more contacts than expected while blue represents less contacts than expected). Aggregated contacts are obtained using the HiContacts::aggregate R function.

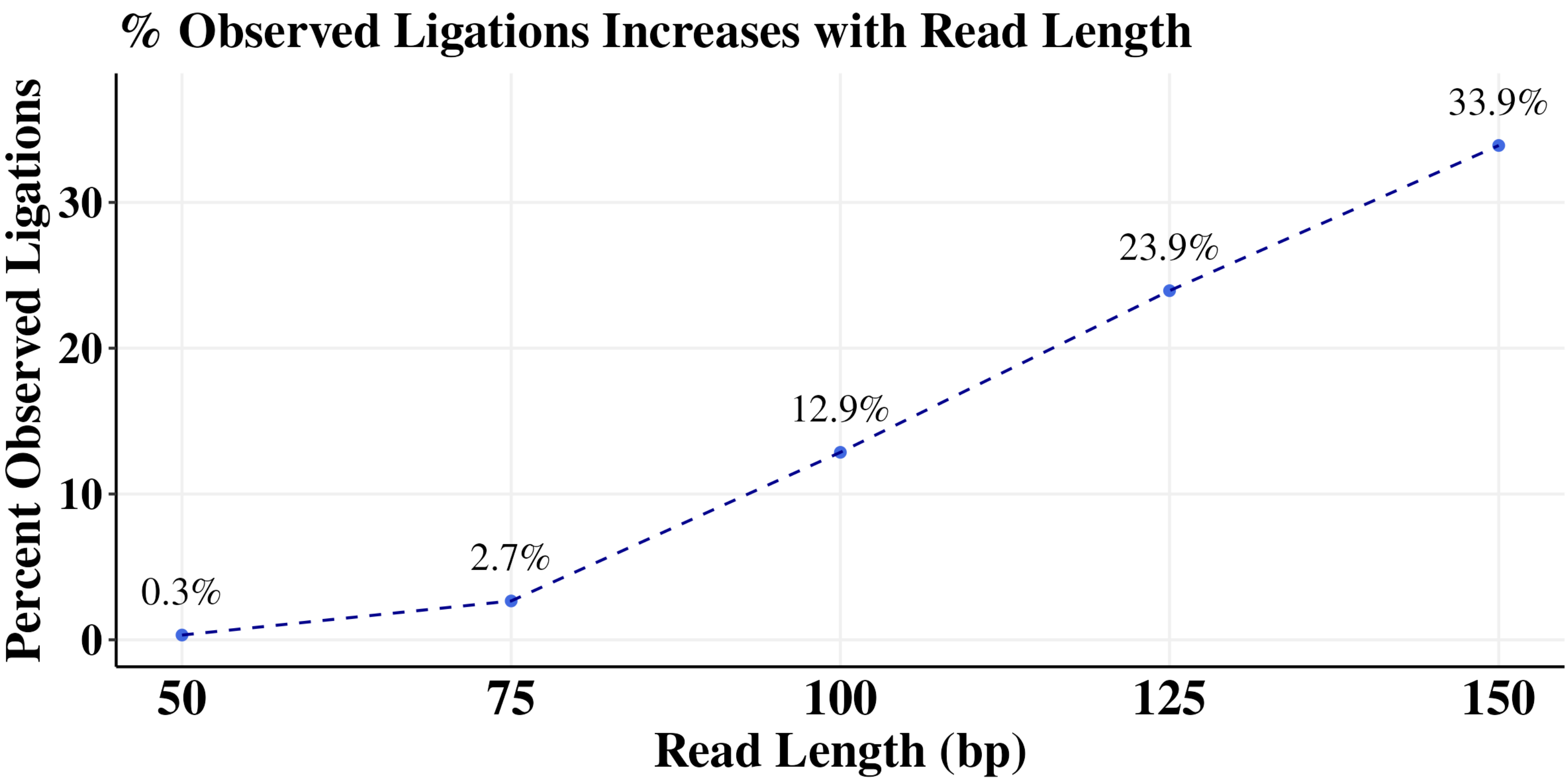

Supplementary Fig. 2. Percent observed ligations increases with read length.

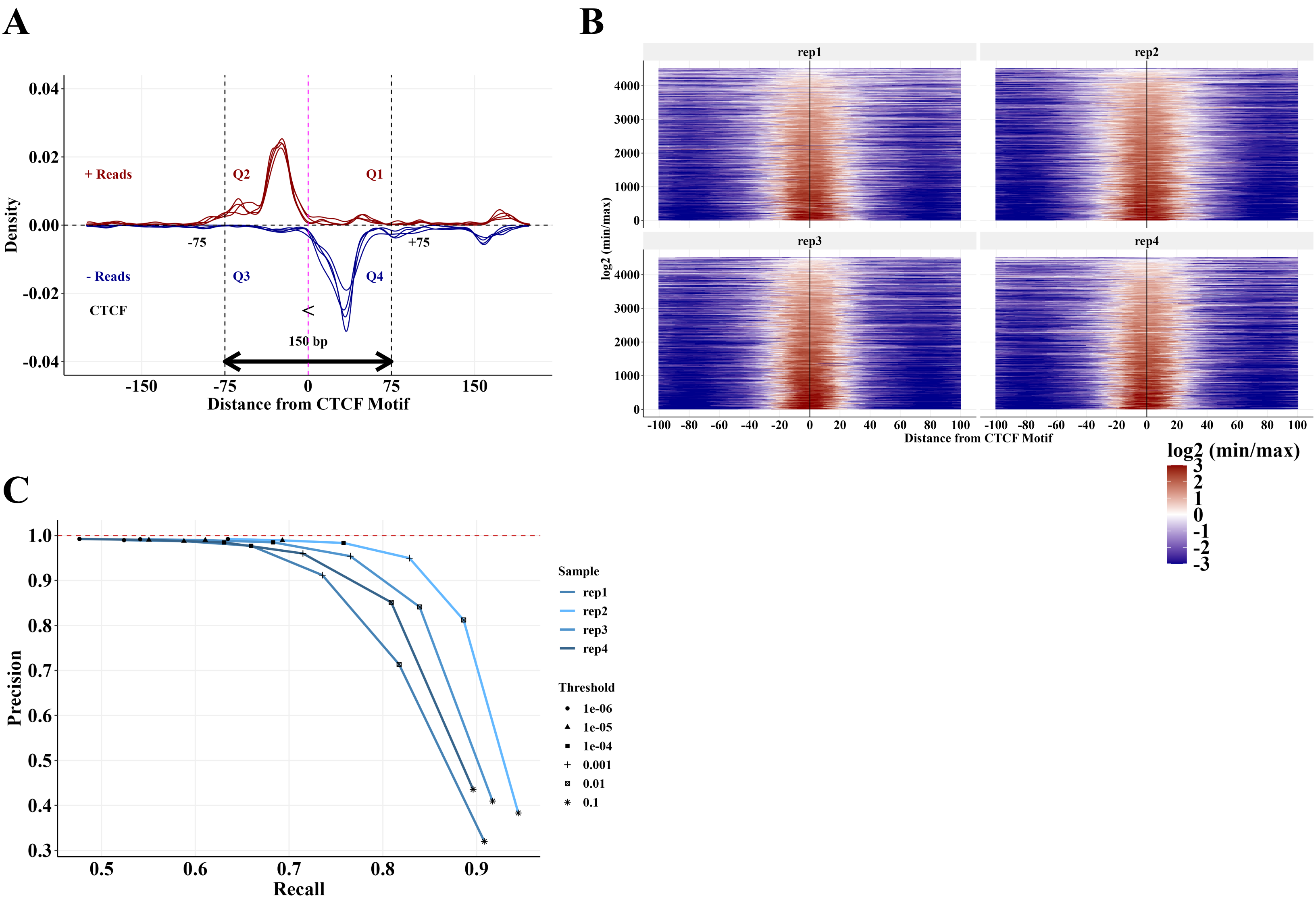

Supplementary Fig. 3. Comparison of K562 CTCF MNase HiChIP replicates. A Fig. 2D read distribution pattern at a CBS is observed across all four replicates. CBS (-) is located at chr1:30,779,763-30,779,781. **B** Fig. 3A CAMEL statistic (log2 (min/max)) pattern observed across all four replicates. **C** Fig. 3B (precision recall curves) by replicate.

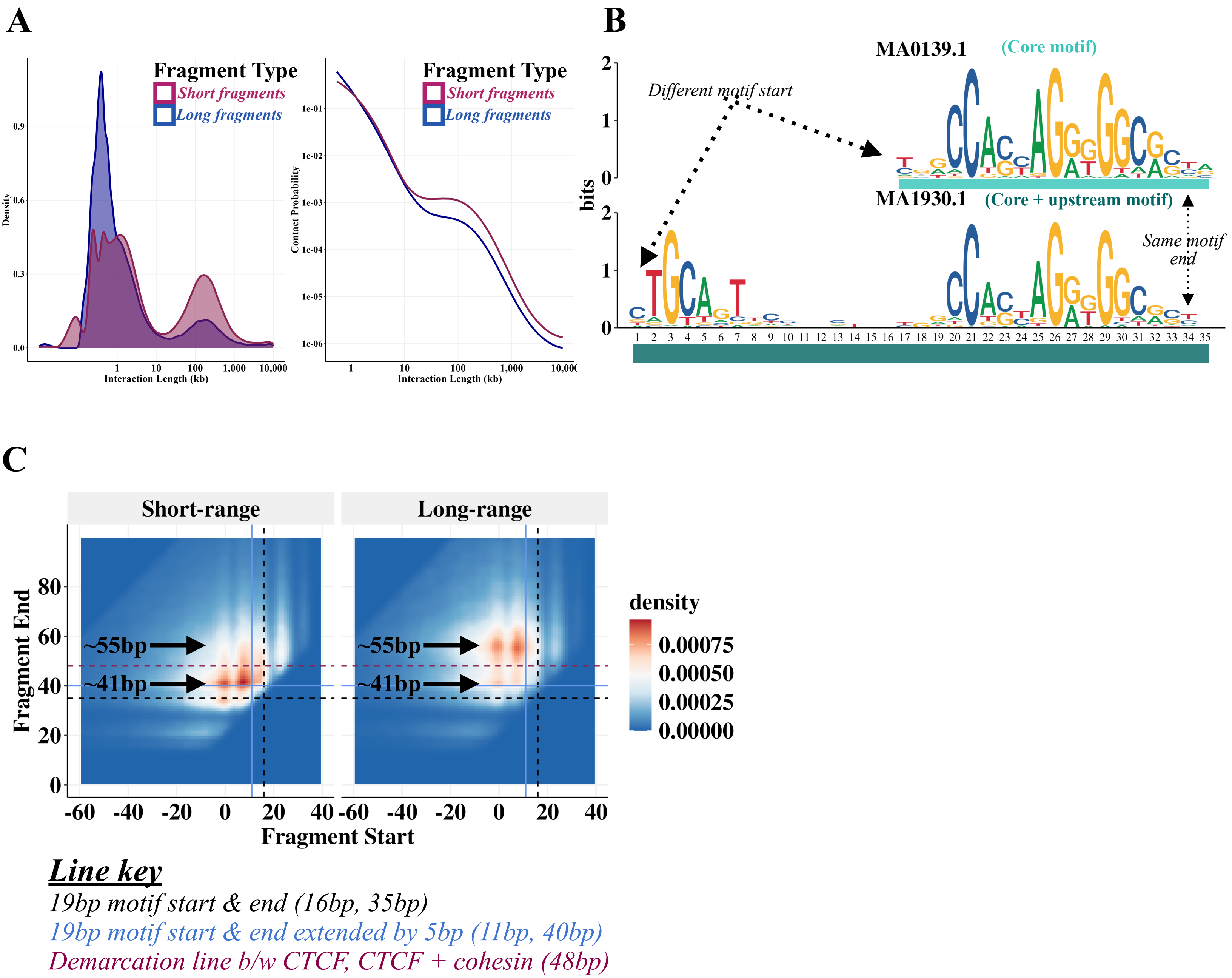

Supplementary Fig. 4. Companion figure to Fig. 4: CTCF and cohesin footprints. A 1D density (left) and P(S) curve (right) of the interaction lengths (kb) of fragments overlapping CBS motifs (+), starting at least 5bp before the motif start, and ending at least 5bp after the motif start. Long and short fragments are defined as greater than or equal to 120bp (long) and less than 120bp (short) respectively. **B** Comparison of JASPAR 19bp (MA0139.1) and 35bp (MA1930.1) CTCF motifs. Meme files (MA0139.1.meme, MA1930.1.meme) were downloaded from JASPAR, read into R (universalmotif::read_meme), and aligned into a ggplot compatible plot (universalmotif::view_motifs). **C** Fig. 4C with annotations. 2D density of all fragments overlapping CBS (+) with fragment length less than 120bp. Fragment end and fragment start are aligned to the start of the long (35bp) CTCF motif (MA1930.1) such that 0 represents the start of the 35bp CTCF motif, 16bp represents the start of the 19bp CTCF motif (MA0139.1), and 35bp represents the end of both CTCF motifs. Annotation lines are added in with explanation in the "Line key", and arrows are drawn to show the locations of the fragment end for the CTCF and CTCF with cohesin footprints.

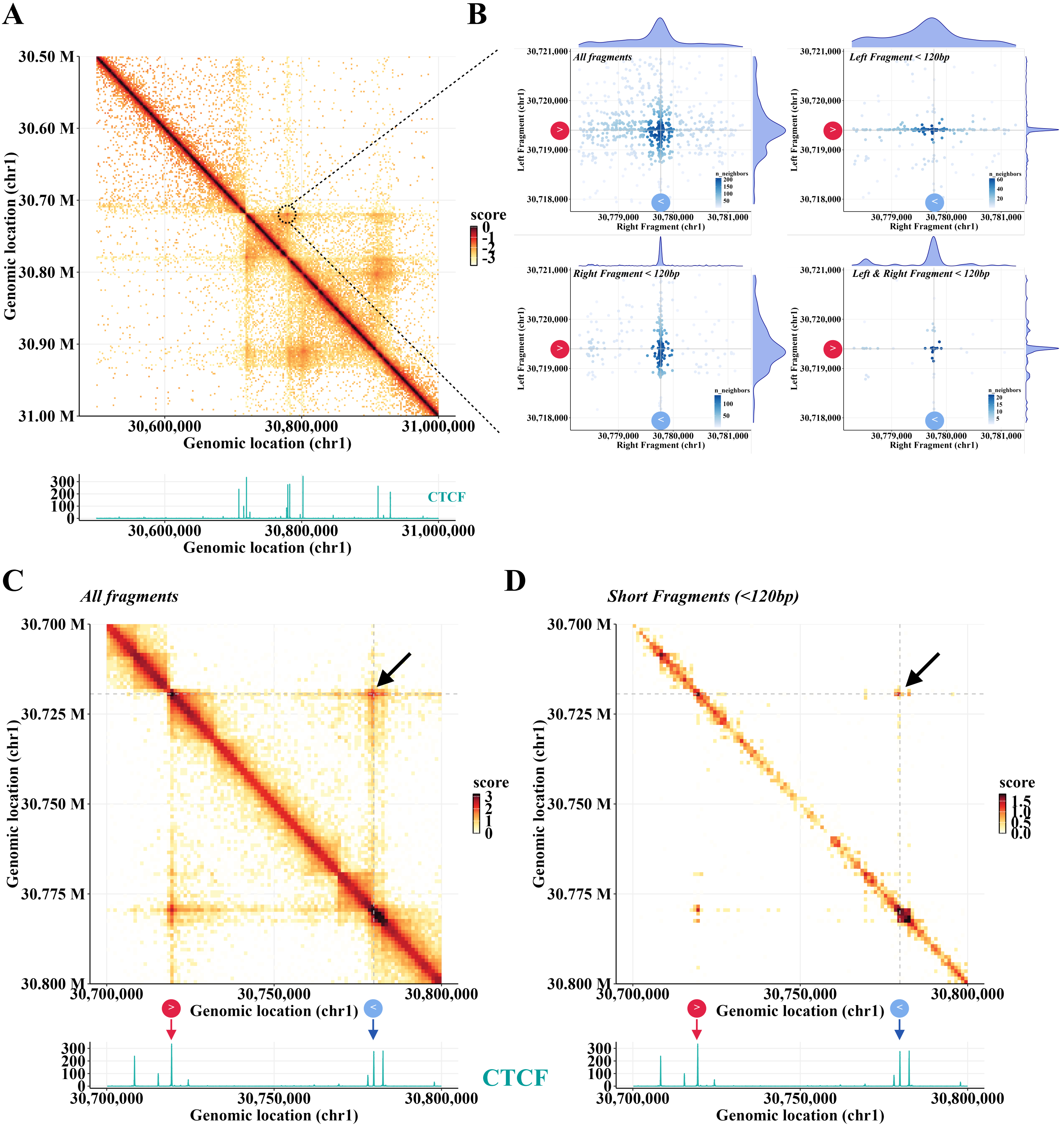

Supplementary Fig. 5. Filtering to short fragments enables identification of CTCF-mediated contacts. A K562 CTCF MNase HiChIP balanced contact map (log10 scale, 2kb resolution) for chr1:30,500,000-31,000,000 with CTCF ChIP-Seq annotation below (ENCFF168IFW). **B** Marginal densities and scatterplots colored by number of neighbors of left fragments vs right fragments (midpoint of fragment) for the loop circled in panel **(A)** between two convergent CTCF motifs: chr1:30,719,390-30,719,408 (+), chr1: 30,779,763-30,779,781 (-). Each panel shows the result of filtering based on fragment length; from left to right top to bottom (also labeled above): all fragments, left fragment < 120bp, right fragment < 120bp, and both left and right fragment < 120bp. The red (>) and blue (<) symbols respectively denote CTCF (+) and CTCF (-) motifs for panels B, C, D. **C** Raw contact map (log10 scale, 1kb resolution) of all fragments for a zoomed in view (chr1:30,700,000-30,800,000) of the loop circled in panel **(A)**, with CTCF ChIP-Seq and motif annotations below. **D** Raw contact map (log10 scale, 1kb resolution) for only short fragments (left fragment and right fragment < 120bp) for the same region as panel **(C)**, with CTCF ChIP-Seq annotation below.

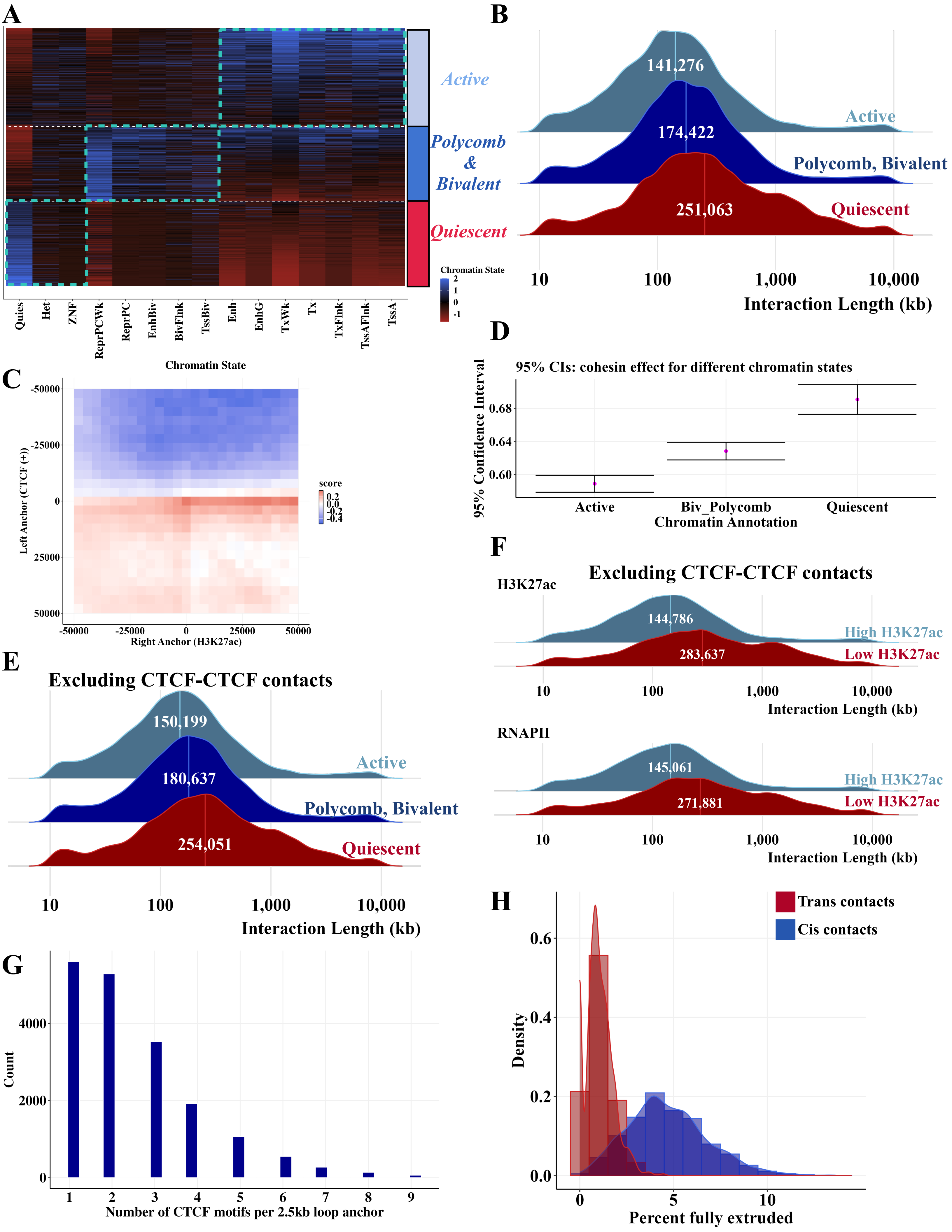
A
Active
Polycomb & Bivalent
Quiescent
Chromatin State
2
1
0
-1
Quies
Het
ZNF
ReprPCWk
ReprPC
EnhBiv
BivFlnk
TssBiv
Enh
EnhG
TxWk
Tx
TxFlnk
TssAFlnk
TssA
Chromatin State
B
141,276
Active
174,422
Polycomb, Bivalent
251,063
Quiescent
10
100
1,000
10,000
Interaction Length (kb)
C
Left Anchor (CTCF (+))
Right Anchor (H3K27ac)
score
0.2
0.0
-0.2
-0.4
-50000
-25000
0
25000
50000
D
95% CIs: cohesin effect for different chromatin states
95% Confidence Interval
0.68
0.64
0.60
Active
Biv_Polycomb
Quiescent
Chromatin Annotation
E
Excluding CTCF-CTCF contacts
150,199
Active
180,637
Polycomb, Bivalent
254,051
Quiescent
10
100
1,000
10,000
Interaction Length (kb)
F
Excluding CTCF-CTCF contacts
H3K27ac
144,786
High H3K27ac
283,637
Low H3K27ac
10
100
1,000
10,000
Interaction Length (kb)
RNAPII
145,061
High H3K27ac
271,881
Low H3K27ac
10
100
1,000
10,000
Interaction Length (kb)
G
Count
4000
2000
0
1
2
3
4
5
6
7
8
9
Number of CTCF motifs per 2.5kb loop anchor
H
Trans contacts
Cis contacts
Density
0.6
0.4
0.2
0.0
0
5
10
Percent fully extruded

Supplementary Fig. 6. Companion figure to Fig. 5: Cohesin extrudes further through quiescent regions than active regions. A ChromHMM annotated 1 MB regions downstream of CBS cluster into three main groups: active, polycomb & bivalent, and quiescent chromatin. Each 1 MB region is characterized by x% TssA (active TSS), y% TssAFlnk (flanking active TSS), z% TxFlnk (flanking transcription), etc. as defined using ChromHMM. Each row corresponds to a CTCF binding site (N = 10,906), and each column shows the normalized fraction of downstream DNA with a specific chromHMM annotation**. B** Ridge plots for the same data graphed in Fig. 5C with averages marked for each chromatin type. This demonstrates how we obtain the estimates reported in Fig. 5F. **C** Metaplot of Intact Hi-C contacts (GEO: GSE237898) aggregated over chromosome one CTCF (+):H3K27ac contacts. All chr1 H3K27ac peaks (ENCODE: ENCFF544LXB) between 10kb and 100kb downstream of CBS (+) are included in this aggregated plot (N = 2732, resolution = 4kb, linear scale, detrended contacts). Contacts are aggregated over all such CTCF:H3K27ac pairwise contacts regardless of whether there is a loop between the CTCF and H3K27ac (to keep this aggregation unbiased). Greater than 10kb was chosen to filter to long-range contacts, and less than 100kb was chosen to keep CTCF:H3K27ac contacts in the same TAD. Red represents more contacts than expected while blue represents less contacts than expected. Aggregated contacts are obtained using the HiContacts::aggregate R function. **D** Controlling for locus-specific variation with linear mixed models does not attenuate the relationship between chromatin state and extruded loop size. **E** Ridge plots for the same data graphed in **(B)** but excluding contacts within 1kb of CTCF motifs on the negative strand (convergent CTCF-CTCF contacts). **F** Ridge plots for the same data graphed in Fig. 5E but excluding contacts within 1kb of CTCF motifs on the negative strand (convergent CTCF-CTCF contacts). **G** Distribution of the number of CTCF motifs in a 2.5kb loop anchor. **H** Estimates of the fully extruded state obtained using trans (red) and cis (blue) contacts. The cis contact density histogram is equivalent to Fig. 5A. Estimate is obtained using left fragments that overlap *CAMEL* identified CBS (+) and have an interaction length greater than 10kb with start and end at least 5 bp from motif start and end, length < 120, and extended fragment end. For each CBS with at least 50 long-range TF-protected fragments overlapping the motif, % convergent is calculated as the number of interaction partners overlapping CTCF (-) motifs / total number of fragments at motif. Because this estimate is conditional on CTCF binding at the anchor, we divide estimates by two to account for the ~50% occupancy of CTCF[33].

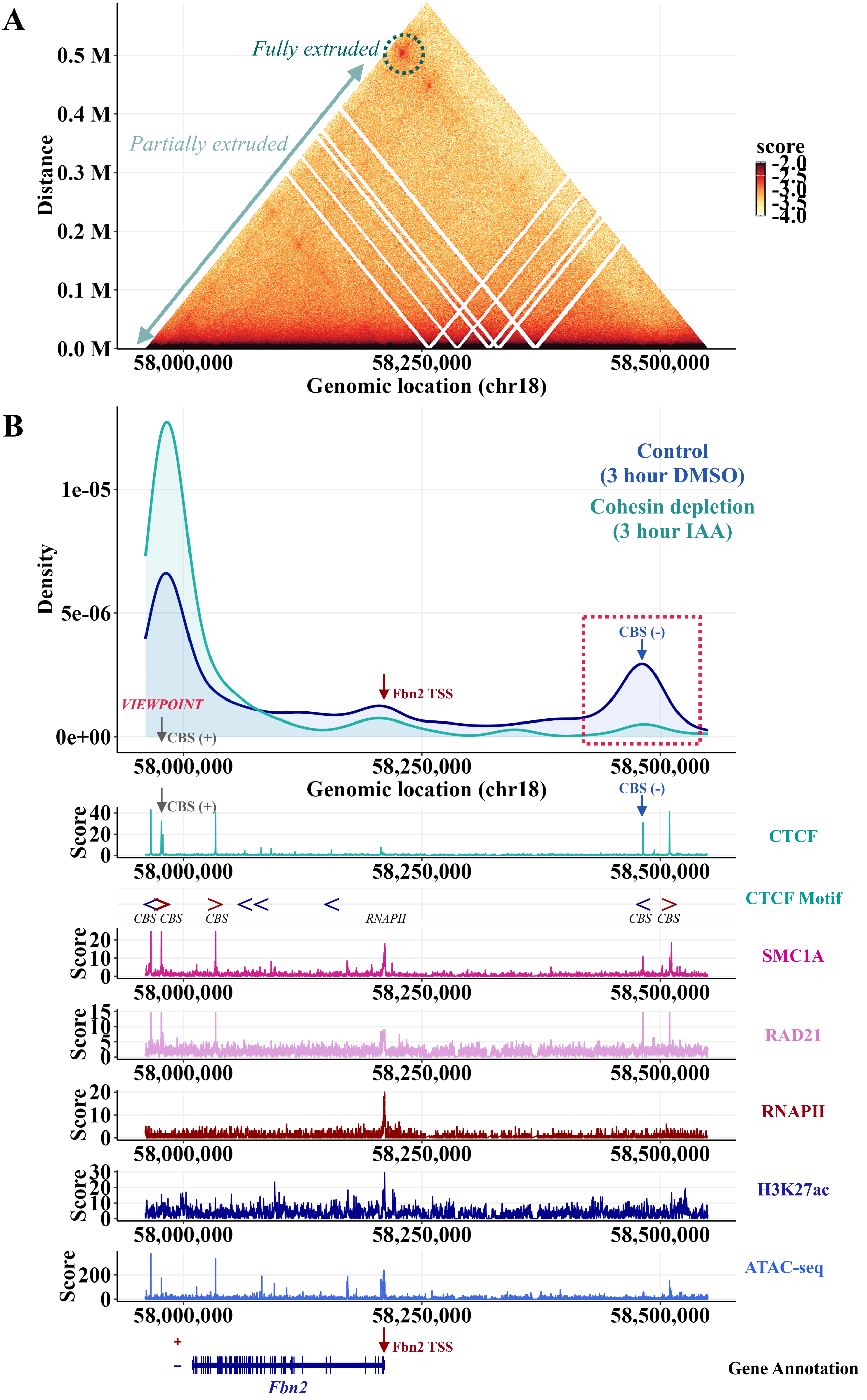
A
Fully extruded
Partially extruded
Distance
0.5 M
0.4 M
0.3 M
0.2 M
0.1 M
0.0 M
score
-2.0
-2.5
-3.0
-3.5
-4.0
58,000,000
58,250,000
58,500,000
Genomic location (chr18)
B
Density
1e-05
5e-06
0e+00
Control
(3 hour DMSO)
Cohesin depletion
(3 hour IAA)
CBS (-)
Fbn2 TSS
VIEWPOINT
CBS (+)
CTCF
CTCF Motif
CBS CBS
CBS
RNAPII
CBS CBS
SMC1A
RAD21
RNAPII
H3K27ac
ATAC-seq
Score
Gene Annotation
Fbn2

Supplementary Fig. 7. mESC *Fbn2* CBS:RNAPII contact lost upon depleting cohesin. A mESC RCMC *Fbn2* TAD balanced contact map (log10 scale, 1kb resolution), visualized with HiContacts::plotMatrix. **B** 1D density for left fragments (<120bp) overlapping the CBS (+) anchoring the left side of the *Fbn2* TAD for control (3 hour DMSO) and cohesin depletion (3 hour IAA). The CBS (+) coordinates are chr18:57,976,797-57,976,815 and the CBS (-) coordinates are chr18:58,481,866-58,481,884 (mm10). The viewpoint (CBS (+)), *Fbn2* TSS, and CBS (-) are marked on the 1D density. ChIP-seq annotations downloaded from GEO and gene annotation (plotted with plotgardener::plotGenes) are included below the 1D density. The GEO accession IDs for the ChIP-seq files are as follows: CTCF (GSM3508478), ATAC-seq (GSE98390), H3K27ac (GSM2417096), RNAPII (GSM6809981), RAD21 (GSE137272), and SMC1A (GSE137272). We use mm10 coordinates for all mESC figures in this paper; files downloaded in other mouse genomes were converted to mm10 using CrossMap.

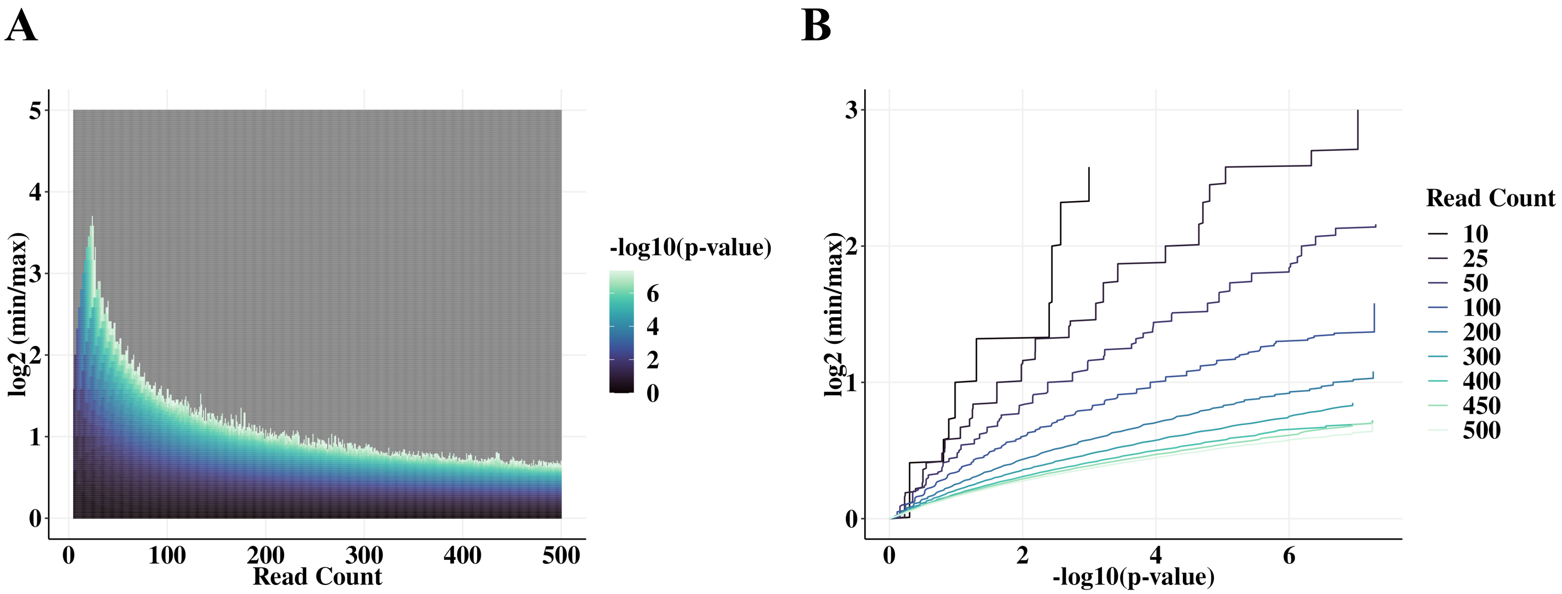

Supplementary Fig. 8. The probability of observing a high CAMEL statistic under the null hypothesis is higher at low read counts. A Simulated log2 (min/max) vs read count colored by -log10(p-value) heat map. **B** Simulated log2 (min/max) vs -log10(p-value) for a few example read counts.

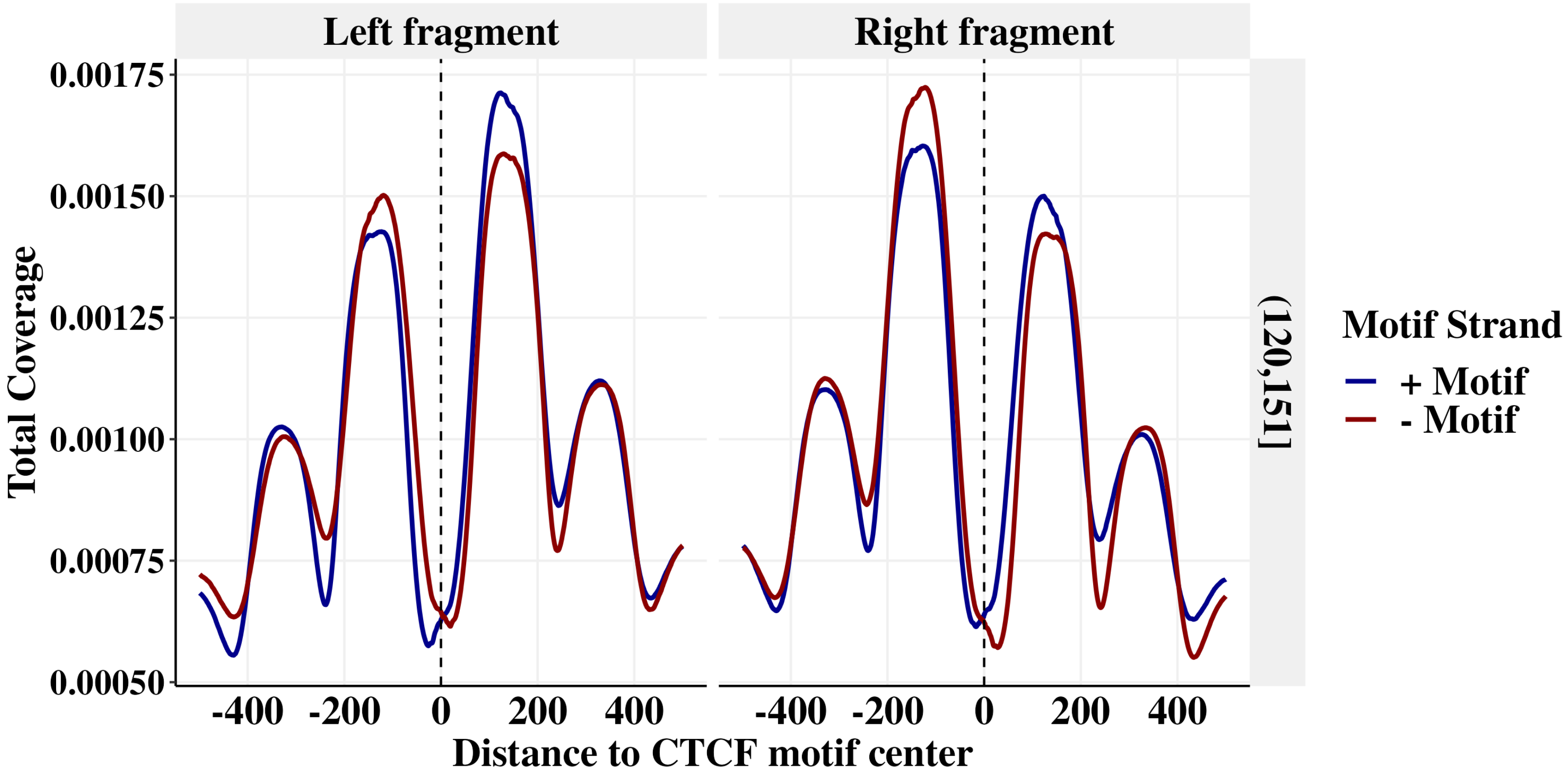

Supplementary Fig. 9. Nucleosomes are preferentially positioned inside the loop. Coverage of long (between 120 and 151bp) fragments faceted by left or right fragment in an interaction pair and colored by the CTCF motif strand. Only fragments with interaction length > 10kb are included.
